# Deeply-sourced formate fuels sulfate reducers but not methanogens at Lost City hydrothermal field

**DOI:** 10.1038/s41598-017-19002-5

**Published:** 2018-01-15

**Authors:** Susan Q. Lang, Gretchen L. Früh-Green, Stefano M. Bernasconi, William J. Brazelton, Matthew O. Schrenk, Julia M. McGonigle

**Affiliations:** 1 School of Earth, Ocean, and Environment, University of South Carolina, Columbia, SC 29208 USA; 2 Department of Earth Sciences, ETH Zürich, Zurich, 8092 Switzerland; 3 Department of Biology, University of Utah, Salt Lake City, UT 84112 USA; 4 Department of Earth and Environmental Sciences, Michigan State University, East Lansing, MI 48824 USA

## Abstract

Hydrogen produced during water-rock serpentinization reactions can drive the synthesis of organic compounds both biotically and abiotically. We investigated abiotic carbon production and microbial metabolic pathways at the high energy but low diversity serpentinite-hosted Lost City hydrothermal field. Compound-specific ^14^C data demonstrates that formate is mantle-derived and abiotic in some locations and has an additional, seawater-derived component in others. Lipids produced by the dominant member of the archaeal community, the Lost City Methanosarcinales, largely lack ^14^C, but metagenomic evidence suggests they cannot use formate for methanogenesis. Instead, sulfate-reducing bacteria may be the primary consumers of formate in Lost City chimneys. Paradoxically, the archaeal phylotype that numerically dominates the chimney microbial communities appears ill suited to live in pure hydrothermal fluids without the co-occurrence of organisms that can liberate CO_2_. Considering the lack of dissolved inorganic carbon in such systems, the ability to utilize formate may be a key trait for survival in pristine serpentinite-hosted environments.

## Introduction

Deep sea hydrothermal systems have long been recognized as important zones of biological activity in the anoxic subsurface, and as potential locations for the evolution of early life on Earth^1,2^. Alkaline serpentinization environments have been proposed as promising locations for the emergence of early life, in part because the high concentrations of H_2_ that are a natural outcome of water-rock reactions can promote the non-biological synthesis of some organic molecules^3^. These environments are widespread in oceanic and continental settings^4^, may have been even more extensive prior to ~3 billion years ago^5^, and may be active on other planetary bodies such as Saturn’s moon Enceledus^6^. While the chemical energy for the synthesis of organic compounds is abundant in serpentinization systems, the relative importance of abiotic vs. biotic processes is unknown. We carried out studies at an alkaline serpentinization system to investigate the abiotic production of organic molecules and to identify how present-day microbial communities interact with these geologically-driven reactions.

The reaction of ultramafic rocks with water results in high concentrations of hydrogen that can provide the geochemical fuel for both biotic and abiotic organic synthesis. Resulting fluids contain high concentrations of reduced carbon compounds such as methane, short chain n-alkanes, and formate that have been proposed to form abiotically based on thermodynamic calculations, laboratory experiments, and geochemical and isotopic measurements^7–15^. Pure hydrothermal fluids can support anaerobic metabolisms such as methanogenesis and sulfate reduction in the absence of mixing with seawater^16–18^.

The oxidized carbon species CO_2_ or its other forms (HCO_3_^−^, CO_3_^−2^) are typically presumed to be the starting material for the abiogenic formation of organic molecules and for primary production. However, oceanic low-temperature alkaline serpentinization environments such as the Lost City Hydrothermal Field, or analogous land-based environments in Oman, California, and Italy, are characterized by extremely low concentrations of dissolved inorganic carbon due to its reduction to hydrocarbons and the rapid precipitation of calcium carbonate at pH above ~9^7,13,19–23^. Thus, the availability of oxidized carbon may be a limiting factor for both abiogenic organic carbon synthesis and for the habitability of serpentinization environments. The organic acid formate has been proposed as an alternative starting material. Benchtop experiments have demonstrated that formate can rapidly form under high H_2,_ reducing conditions^11^, and that certain abiotic synthesis reactions proceed more readily when formate is used as a starting material^24,25^.

The Lost City Hydrothermal Field is an iconic example of a low-temperature serpentinization system, and fluids there have elevated formate concentrations (36–158 µM) that have been proposed to form abiotically, through equilibration^14^:

1 $${{{\rm{HCO}}}_{3}}^{-}+{{\rm{H}}}_{2}={{\rm{HCOO}}}^{-}+{{\rm{H}}}_{2}{\rm{O}}$$

Formate has also been proposed to form abiotically in the lower pH system of Mid-Cayman Rise, but only after mixing of the hydrothermal fluids with local seawater^26^. If mixing is also required for formate production at Lost City, neither it nor ∑CO_2_ (sum of CO_2aq_, H_2_CO_3_, HCO_3_^−^, and CO_3_^−2^, used to distinguish from the chemically identical but isotopically distinct seawater dissolved inorganic carbon) would be available as a starting material for abiotic synthesis or primary production during much of the hydrothermal fluid pathway, potentially limiting the importance of alkaline serpentinization in the development of early life.

We carried out a series of ^13^C and ^14^C analyses to identify the origin of formate at Lost City and to determine if this compound represents an important carbon source to the dense microbial communities that inhabit the Lost City chimneys. The anoxic interior of chimneys are dominated by a single archaeal phylotype, the Lost City Methanosarcinales (LCMS)^27,28^. Endmember fluids retain 1–4 mM sulfate that co-exists with hydrogen, and geochemical trends across the field indicate microbial sulfate reduction is an important subsurface process^29,30^, possibly carried out by organisms related to *Desulfotomaculum*^28,31^. Chimney exteriors host organisms involved in the oxidation of sulfur and CH_4_ (e.g. *Methylomonas*, *Thiomicroscopira*)^28^.

## Results and Discussion

**Figure 1:**
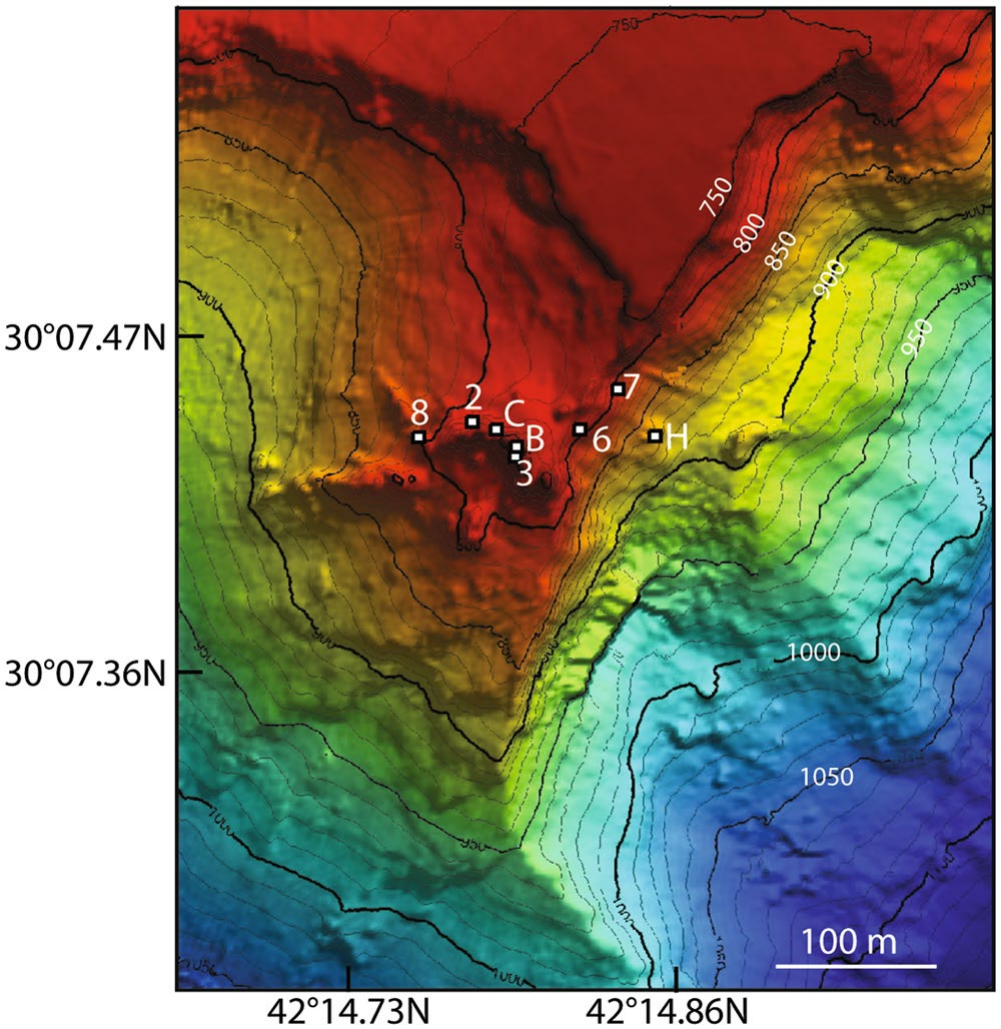
Bathymetric map of the the Lost City hydrothermal field with individual vent locations identified by their Marker ID. Figure is from Kelley *et al*., 2005 [ref.^21^] and is not covered by the CC-BY license. Reprinted with permission from AAAS.

**Figure 2:**
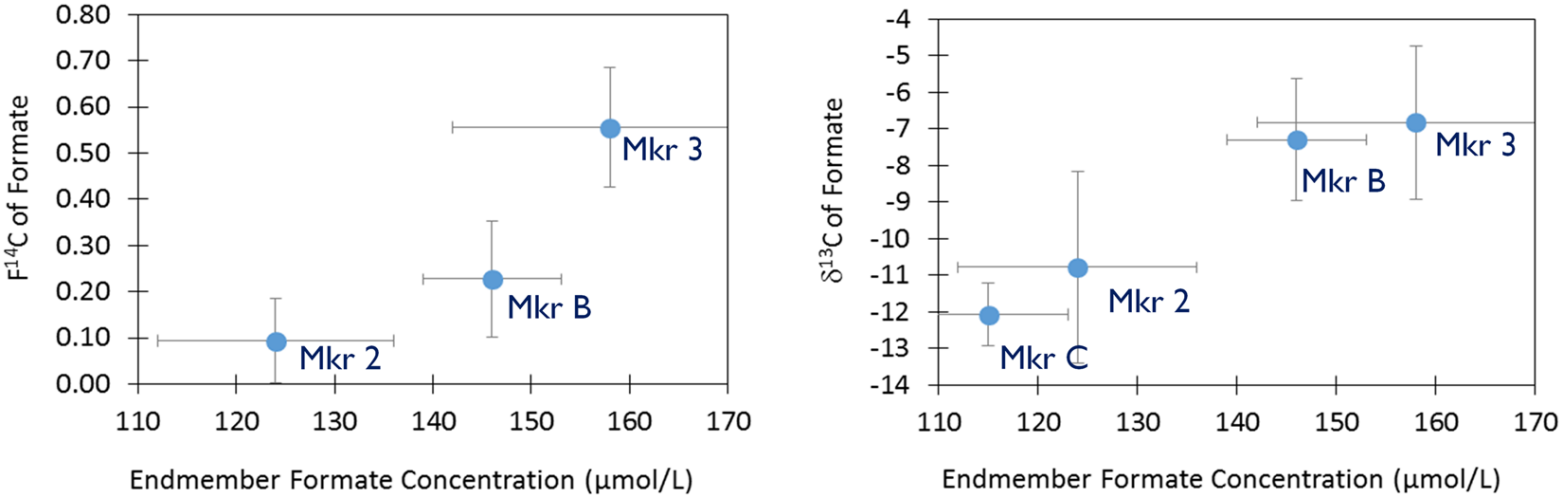
Average (**a**) F^14^C of formate vs. formate concentration and (**b**) δ^13^C of formate vs. formate concentration in different vents. Error bars represent the standard deviation of multiple samples (N = 2–6; full data in Supplemental Table 1). The amount of formate isolated from Marker C samples was insufficient for ^14^C analysis.

The isotopic signature (^13^C, ^14^C) of formate was determined for 6 fluid samples collected from Lost City chimneys at 4 locations (Markers 2, C, B, 3; Fig. 1, Table 1 and Supplemental Table 1). Formate from Marker 2 lacks radiocarbon (F^14^C = 0.09 ± 0.09), demonstrating that it is ultimately derived from ^14^C-free carbon (Fig. 2; Supplemental Table 1). It eliminates the fermentation or degradation of chimney biomass as a possible source, as chimney total organic carbon has F^14^C values ranging from 0.480 to 0.888 (Table 1)^30^. The stable isotope signatures of formate from both Marker 2 and Marker C (−13.0 to −8.9%) overlap with those of ^14^C-free methane (−13.6 to −9.3%) and short-chain hydrocarbons (−16.9 to −13.1%)^13^. Mantle CO_2_ is inferred to be the starting material for C_1_ + alkanes; although this CO_2_ is subsequently removed from the fluids due to abiotic reduction to organic compounds and/or precipitation as carbonate. Input of mantle volatiles is also evident from elevated ^3^He/^4^He ratios^13^. The lack of ^14^C in the formate indicates it is also formed from mantle CO_2_. Temperatures in the subsurface of Lost City (~180 °C^32^) where this starting material would be available are sufficiently high to promote its abiotic conversion to formate (>175 °C)^10^ and above the current temperature limit to life (122 °C^33^). In contrast, formate from Markers B and 3 was present in higher concentrations, was somewhat modern (F^14^C = 0.14–0.56), and had more positive δ^13^C values (−9.1 to −4.3%) (Fig. 2; Supplementary Table S1). The more modern formate at Markers B and 3 points to an additional carbon source in these locations.

**Table 1:** Concentrations and isotopic compositions of known carbon species in the Lost City environment. n.d. is not determined.

Species	Concentration	δ^13^C	F^14^C
	(µmol/L)	(%_0_ vs VPDB)	
*Constituents dissolved in fluids*			
CH_4_^13,21^	890–1980	−13.6 to −9.3	0.002 to 0.006
C_2_H_6_ - C_4_H_10_^13^	0–1.8	−16.9 to −13.1	n.d.
Formate^14^^ ,present work^	36–158	−12.1 to −6.8	0.09 to 0.56
Acetate^14^^ ,present work^	1–35	−26.6 to −20.3	n.d.
Total hydrolizable amino acids^67^	0.7–2.3	n.d.	n.d.
Mantle CO_2_ ‘predicted’ to be in endmemeber fluids^a^	2000–4100	−12 to −2	0
Dissolved ΣCO_2_ in endmemeber fluids from Mkrs C, 2, B^13^	0.1–1 (avg 0.2)	≈ −9	n.d.
Dissolved ΣCO_2_ in endmemeber fluids from Mkr 3^13^	10–26	≈ −9	n.d.
Dissolved inorganic carbon in seawater (800 & 550 m)^13^	2200	0.8	0.993 to 1.052
*Constituents in actively venting chimneys*			
Calcium Carbonate^21,23^	n/a	−7 to + 13	0.957 to 1.002
Total organic carbon^21,53^	400–1500 ppm	−12.4 to −3	0.480 to 0.888
Total hydrolizable amino acids^67^	n.d.	−18.3 to + 8.7	n.d.
Bacterial lipids (fatty acids, non-isoprenoidal diethers)^37,42^	0–2.4 µg lipid/g chimney	−31 to −1.1	n.d.
Archaeal lipids (isoprenoidal diethers, PMIs)^37,42^	0–3.5 µg lipid/g chimney	−12 to + 24.6	n.d.
Eukaryotic lipids (polycyclic terpanoids)^37,42^	0–0.5 µg lipid/g chimney	−28.5 to −15.4	n.d.
Mono-unsaturated fatty acids, >95% C16:1 and C18:1^b^		−18.7	0.68 ± 0.03
Saturated, straight-chain fatty acids, >99% C16:0^b^		−8.9	0.13 ± 0.02
Combined Pentamethylicosanes^b,c^		1.7	0.24 to 0.49
Squalene^b^		−11.0	0.17 ± 0.02
Phytanic Acid^b^		−1.4	0.22 ± 0.02

^a^Concentrations based on relationship between CO_2_ and ^3^He ref.^13^. Isotopes based on fluid inclusions from gabbros and olivine gabbros ref.^68^.

^b^From Marker 7 chimney only; present work.

^c^PMIs could not be fully separted from a UCM hump which contributed up to 20% of the peak area by GC-MS. Reported range reflects mass balance calculation assuming the F^14^C of the total PMI + UCM fraction (F^14^C = 0.39 ± 0.02) had either a fully modern (F^14^C = 1.0) or fully dead (F^14^C = 0) signature.

The relative concentrations of formate and ΣCO_2_ in Lost City fluids suggest they are not controlled by purely equilibrium processes. In almost all locations at Lost City, formate is present in far higher concentrations than expected for the amount of ΣCO_2_ present (ref.^14^; Supplemental Materials). The one exception is at Marker 3 where the two species are close to equilibrium due to ΣCO_2_ concentrations that are more than an order of magnitude higher than those at other vents (10–26 versus 0.1–0.6 µmol/L)^13^. Fluids from Marker 3 have been influenced by near-surface mixing with seawater that is the likely source of the additional inorganic carbon^14,34^.

In the simplest scenario, formate has two sources: an abiotically synthesized, mantle-derived, F^14^C free source with a more negative δ^13^C signature and an additional source derived from modern inorganic carbon in seawater (F^14^C = 0.993–1.052^13^) and a more positive δ^13^C signature. By using the average endmember concentration and F^14^C and δ^13^C of formate at each vent, it is possible to calculate the concentrations of formate that are derived from each of these two endmembers. The data from Markers C, 2 and B could be accounted for with a constant contribution of a ^14^C-free endmember with a concentration of ~112 ± 20 µmol/L and a δ^13^C value of −12.7 ± 3.8%, plus contributions of a modern endmember with an isotopic signature of F^14^C = 1.02 ± 0.04 and δ^13^C =  + 8.4 ± 1.6% and a concentration that varies between locations, from 3–40 µmol/L. The derivation of these values is described in full in the Supplemental Materials.

The calculated concentration of formate that is radiocarbon-free at Marker 3 is significantly less (72 ± 8 µmol/L), while the calculated concentration of modern-derived formate is significantly more (~90 µmol/L) than in the other chimneys. This apparently lower contribution of ^14^C-free formate at Marker 3 is remarkable given that its total concentration is higher than the other locations. When combined with the observation that the concentrations of formate and ΣCO_2_ are close to equilibrium in this location, the F^14^C data point to a rapid equilibration of both concentrations and isotopes between these two species at Marker 3. This equilibration is, presumably, due to biological processes as the abiotic equilibration between the two is expected to be exceedingly slow at temperatures <175 °C^10^ and fluid temperatures are 88 °C^21^. In contrast, the biological conversion between CO_2_ and formate is rapid and reversible^35,36^. Therefore, microbial communities living along the fluid pathway of Marker 3 may be rapidly converting between the mantle-derived, ^14^C-free-formate carried with pure endmember fluids and the inorganic carbon that is present in the seawater that has been entrained. If true, the ^14^C of the ΣCO_2_ from Marker 3 should be a similar mixture of modern and dead carbon. Unfortunately, it has not been possible to make this measurement due to low fluid concentrations^13^. This same process likely occurs at other locations but is less evident due to a lower amount of seawater entrainment.

To investigate whether the microbial communities inhabiting the carbonate chimneys are actively utilizing formate, we determined the ^14^C content of lipid biomarkers from a Lost City chimney (Marker 7) that could be attributed to specific microbial communities. Previous work has demonstrated that bacterial fatty acids have a bimodal ^13^C distribution in many chimneys, with fully saturated C16:0 and C18:0 having more positive isotopic signatures than unsaturated molecules (e.g. −25.4 to −16.8 versus −9.1 to −1.1 respectively at Marker 7)^37^. This pattern has been attributed to a distinction between bacteria inhabiting the interior of the chimney where seawater DIC availability is limited and those living on the exterior of the chimney where it is abundant^37^. In agreement with this interpretation, the saturated C16:0 fatty acid lacks ^14^C while, in contrast, the mono-unsaturated fatty acids C16:1 and C18:1 have a mixed F^14^C signature of 0.68 ± 0.03 (Table 1). This result demonstrates that bacteria living in the anoxic interior of the chimney metabolize a ^14^C-free carbon source that is consistent with their utilization of formate. Mantle CO_2_ is also ^14^C-free but multiple lines of evidence indicate it is stripped from fluids in the subsurface: (a) similar to other alkaline serpentinization systems^15^, Lost City endmember fluids lack significant concentrations of any ∑CO_2_ (0.1–0.6 µmol/L, with the exception of Marker 3 where seawater inputs raise concentrations to 10–26 µmol/L; Table 1)^13^ due to the rapid precipitation of calcium carbonate in the subsurface^22,33^ (b) calcium carbonate from actively venting chimneys has modern ^14^C signatures, indicating that it is precipitated from seawater bicarbonate, and that mantle CO_2_ is not available (Table 1)^21,23,30^.

Metagenomic data from two Lost City chimneys (Marker 3, Marker 5) are also consistent with the ability of some chimney microorganisms to metabolize formate. The enzyme formate dehydrogenase catalyzes the reversible oxidation of formate to CO_2_^38^. Several sequences of the alpha subunit of formate dehydrogenase (*fdhA*) were detected in the chimney metagenomes; the closest bacterial database matches were with sequences related to methylotrophic and sulfate-reducing bacteria (Table 2). The most abundant bacterial *fdhA* sequences in both metagenomes were most similar to *fdhA* from *Desulfitibacter alkalitolerans*, an alkalitolerant sulfite-reducing member of family Peptococcaceae (order Clostridiales) that can use formate as an electron donor^39^. However, the Lost City sequences were still quite divergent from any previously reported *fdhA* genes (only 50–74% amino acid identities with *Desulfitibacter alkalitolerans*), and they could not be placed on a phylogeny of *fdhA* with high confidence.

**Table 2:** Metagenomic sequences predicted to encode the alpha subunit of formate dehydrogenase (fdhA) in Lost City chimneys, as defined by KEGG protein function K00123.

Location	Contig ID	Start - Stop	Best Match	Identities	Metagenome Coverage
Marker 3	3862contig-30000000	319–1782	ANME-2 metagenome (CAI64341.1)	50%	80.74
Marker 3	3862contig-19000000	620–919	*Desulfitibacter alkalitolerans* (WP_028308877.1)	50%	78.59
Marker 3	3862contig-7000000	76–252	*Methylophaga muralis* (WP_069296139.1)	89%	25.59
Marker 5	H08contig-3000000	224–1687	ANME-2 metagenome (CAI64341.1)	50%	107.85
Marker 5	H08contig-0	245–823	*Desulfitibacter alkalitolerans* (WP_028308876.1)	74%	72.38
Marker 5	H08contig-0	959–1291	*Desulfotalea pyschrophila* (WP_041277585.1)	55%	72.38
Marker 5	H08contig-8000000	101–232	*Thalassobius mediterraneus* (WP_058317529.1)	86%	12.21

Previous studies have detected 16 S rRNA gene sequences similar to *Desulfotomaculum alkaliphilum* in Lost City chimneys and fluids^28,40^. *Desulfotomaculum alkaliphilum*, like *Desulfitibacter alkalitolerans*, is also a member of the family Peptococcaceae and is thermophilic, alkaliphilic and a sulfate-reducer capable of growth on formate^41^. *Desulfotomaculum fdhA* genes also show similarity to the Lost City bacterial *fdhA* sequences described above, and *dsrA* (alpha subunit of dissimilatory sulfite reductase, a diagnostic gene for sulfate reduction) sequences similar to *Desulfotomaculum* genes are also abundant in the Lost City chimney metagenomes. Therefore, these results suggest that sulfate reduction at Lost City is most likely to be mediated by *Desulfotomaculum*-like bacteria and that these organisms may be fueled by formate instead of or in addition to hydrogen.

The archaeal community in the chimneys is dominated by the Lost City Methanosarcinales (LCMS), to the point that they are the only detectable archaea in some locations^27,40^. Unusually positive δ^13^C values of lipid biomarkers that are exclusively produced by methane-metabolizing archaea, pentamethylicosane and its unsaturated related compounds (PMIs), have been attributed to carbon limitation by methanogens consuming dissolved inorganic carbon^37,42^. Attempts to fully isolate the PMIs for ^14^C analysis were unsuccessful as they co-elute with a minor unresolved complex mixture (UCM) hump. The total F^14^C value of the combined isolate was F^14^C = 0.39 ± 0.02 (Table 1). Mass balance calculations assuming the UCM is either entirely modern or entirely dead require the PMIs to have a F^14^C value of 0.24–0.49. Phytanic acid and squalene have also been detected in the carbonate chimneys and tentatively attributed to the same archaeal community based on their δ^13^C signatures, though contributions from bacterial or eukaryotic sources cannot be ruled out^42^. These compounds have F^14^C values that are 0.22 ± 0.02 and 0.17 ± 0.02, respectively (Supplemental Table 2). The mixed ^14^C signature of PMIs rules out that the LCMS depends entirely on seawater dissolved inorganic carbon or materials, such as the calcium carbonate of the chimneys, derived from seawater DIC. Instead, it must rely on a carbon source with a substantial mantle-derived component but, given the mixed ^14^C signature, it is also unlikely that they solely consume methane. Their anomously positive δ^13^C values also point to carbon limitation and are not consistent with a reliance on acetate (δ^13^C_acetate_ = −27 to −20% across the field; Table 1) or anaerobic oxidation of methane^37^.

Archaeal formate dehydrogenase sequences of the *fdhA* enzyme were also identified in the metagenomic data (Table 2). To investigate whether these represent methanogenic archaea utilizing formate as a carbon source, a phylogeny of genes from methanogens encoding *fdhA* sequences was constructed (Supplementary Figure 2). Neither of the two archaeal *fdhA* sequences in Table 2 showed close phylogenetic relationships with *fdhA* genes from methanogens known to use formate as a carbon source. The predicted archaeal *fdhA* sequence from Marker 3 was highly divergent from all known *fdhA* sequences and was not included in Supplementary Figure 2.

The second archaeal *fdhA* sequence from Marker 5 (large bold font in Supplementary Figure 2) belongs to a divergent clade of *fdhA* that includes two species of *Methanolobus* that can only use methylated compounds as methanogenic substrates and a metagenomic sequence from an enrichment of ANME-2 anaerobic methanotrophic archaea. (ANME-2 and *Methanolobus* species belong to the same order as the Lost City Methanosarcinales.) These four *fdhA* sequences form a clade that is clearly distinct from any *fdhA* sequences from known formate-utilizing or hydrogenotrophic methanogens (Supplementary Figure 2).

The presence of *fdhA* is necessary, but not sufficient, to enable formate-based methanogenesis. Many methanogens that contain *fdhA* are not able to use formate as a carbon source (Supplemental Figure 2). The inability of many of these methanogens to utilize formate is mysterious, but the gene *fdhC*, which is predicted to encode a transporter protein, is thought to be necessary for transport of formate into the cell^43^. A search for *fdhC* in the Lost City chimney metagenomes, recovered only proteins similar to *Thiomicrospira* transporters that are more likely to be transporters of nitrite, not formate. No *fdhC* sequences belonging to methanogenic archaea were detected. The absence of genes in metagenomic data must always be interpreted with caution, but the lack of *fdhC* sequences in this dataset supports the interpretation of the *fdhA* phylogeny that Lost City Methanosarcinales archaea are unlikely to directly utilize formate from the environment.

Ideally, all three lines of evidence presented here would have been carried out on samples from the same suite of Marker locations. The vast majority of samples from Lost City have been previously consumed during earlier analyses. Here we have applied techniques that have been developed in the >12 years since these samples were collected to the few that remained in our laboratories. In many cases, however, extrapolating between locations is justified by results from previous studies. For example, while the lipid ^14^C data is solely from Marker 7, previous work indicates this location has fatty acid and archaeal lipids with similar concentrations, distributions, and ^13^C signatures similar to Markers 2, 3, B, H, and C^37,42^. Similarly, while the metagenomic data is from Markers 3 and 5, 16 S rRNA data indicates the dominance of the same Lost City *Methanosarcinales* phylotype in all analyzed actively venting chimneys (Markers 2, 3, H, C)^28^.

**Figure 3:**
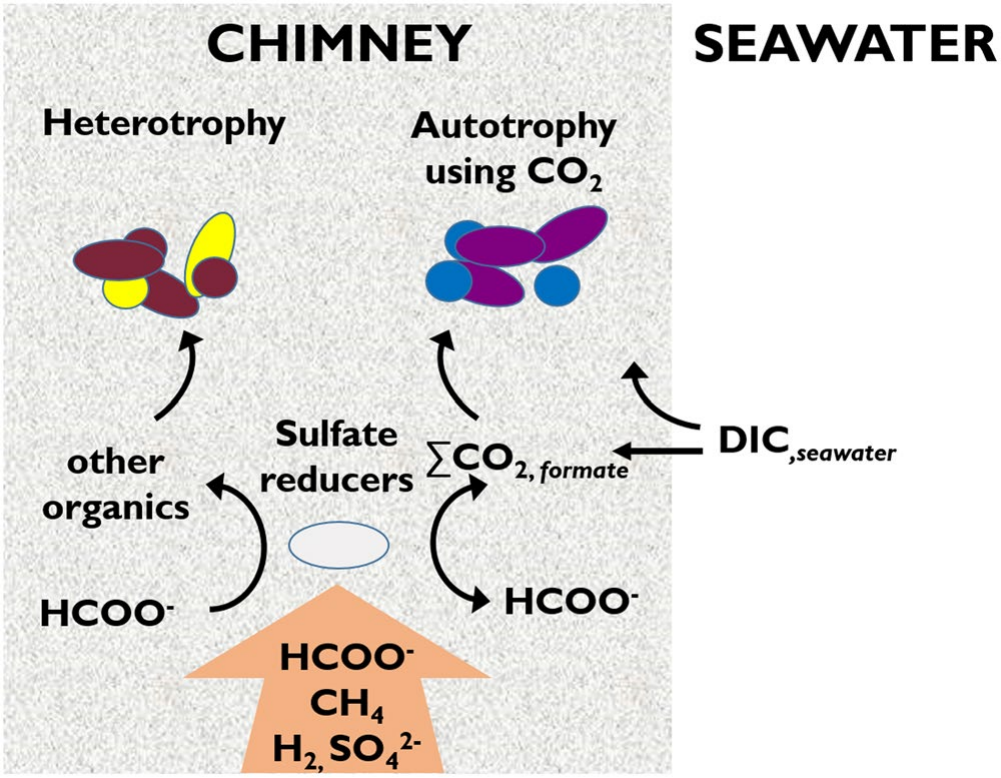
Schematic of proposed carbon-microbe relationship in Lost City chimneys. Anoxic hydrothermal fluids transport ^14^C-free formate, methane, hydrogen, and sulfate through carbonate brucite chimneys. Sulfate reducers convert formate to ΣCO_2_ that can then be utilized by autotrophs such as the Lost City Methanosarcinales. Depending on location, some seawater dissolved inorganic carbon is also incorporated into the DIC pool and is also available for microorganisms living in the chimneys. Due to the reversibility of the formate dehydrogenase enzyme, this modern carbon can be incorporated into the formate pool.

The picture that emerges is a system in which abiotic formate is consistently supplied with pure hydrothermal fluids and utilized by sulfate reducing bacteria who, in part, convert it to ∑CO_2_ (Fig. 3). This CO_2_ would briefly be available to other members of the anaerobic microbial community, including the Methanosarcinales. In near-surface regions the ∑CO_2_ pool would mix with minor inputs of modern seawater DIC, either from entrainment from seawater or the minor dissolution of the chimneys themselves. The mixed ^14^C signature of the PMIs could therefore be accounted for by the Methanosarcinales consuming CO_2_ liberated during the activity of the formate dehydrogenase gene. This interpretation is consistent with a recent study that identified *Firmicutes* as the first inhabitants of juvenile and nascent chimneys in an analogous alkaline serpentinization environment (Prony Bay in New Caledonia) while methane cycling archaea are only identified in older, more established chimneys^44^.

Serpentinization systems have been increasingly proposed as locations where early metabolic pathways could have emerged on Earth and other planets, and they are also critical to understanding the extent and activity of life below the oxygenated surface of Earth. Abundant geochemical energy is available to drive abiotic synthesis and/or microbial metabolisms but the lack of dissolved inorganic carbon may limit the reactions that can proceed. The last universal common ancestor (LUCA) has been proposed to be an anaerobic autotroph similar to methanogens and clostridia, living off of H_2_, CO_2_, and N_2_ in an environment such as Lost City^45^. The core metabolic reactions of LUCA may have evolved as mimicry of geochemical reactions that result in the abiotic synthesis of organic compounds, which still occur in the subsurface of Lost City today. Our results complicate this scenario somewhat by indicating that the methane-cycling archaea presently inhabiting this environment do not have ready access to dissolved CO_2_ and, instead, rely on the biological conversion of formate.

## Materials and Methods

### Sample Collection

Fluids from eight actively venting carbonate-brucite chimneys across the Lost City hydrothermal field were collected in 2003 by DSV Alvin using previously described ‘major’ samplers^46^ and the Hydrothermal Fluid and Particulate Sampler (HFPS)^47^. These samplers are well-suited for the analysis described here because they have low organic carbon blanks when properly cleaned^48^. Both filtered (*in situ*) and unfiltered HFPS samples were collected; filters used *in situ* were either glass fiber filters (Whatman GF/F; combusted at 500 °C for 5 h) or 0.2 µm nitrocellulose filters (Millipore). Upon arrival on deck, subsamples for organic analyses were stored in 100 mL glass vials (combusted at 500 °C, 5 h) with acid-washed Teflon lined caps (10% HCl, overnight); samples were then stored frozen (20 °C) until analysis on shore.

The chimney samples used for isotopic and metagenomic analyses were collected during two cruises. Sample 3862–1325 was collected from Marker 3 in 2003 by DSV Alvin aboard the R/V Atlantis. Samples H08_080105_Bio5slurpB1, from Marker 5, and H02_072605_Bio1slurpA2_0354, from Marker 7, were collected in 2005 during a National Oceanic and Atmospheric Administration (NOAA) Ocean Explorer cruise with the ROV Hercules aboard the R/V Ronald H. Brown. In all cases, samples were immediately placed in a sterile Whilr-Pak® sample bag upon arrival on deck and stored at −80 °C until analysis. DNA was extracted from the Marker 3 and Marker 5 samples according to a previously published protocol^49^. Lipids were extracted from the Marker 7 sample as described below. Geochemical analysis of sample 3862-1325 was previously published^50,51^. No previous microbiological analyses of this sample have been published, but a similar sample collected from Marker 3 during the same expedition (3881-1408) was the subject of previously published DNA sequencing studies^28,40^. The concentrations and ^13^C signatures of lipids, DNA fingerprinting with TRFLP (terminal restriction fragment length polymorphism), and quantification with qPCR (quantitative polymerase chain reaction), were previously described for samples H02_072605_Bio1slurpA2_0354 and LC_H08_080105_Bio5slurpB1 in ref.^42^, where they were referred to as LC-7a and LC-5, respectively.

### Compound Specific Isotope Analysis of Organic Acids

The isotopic signatures of formate and acetate were determined with the method of^52,53^. Briefly, the method consisted of the following steps: (1) Apolar material is removed from 5–15 mL samples by passing them over a C18 SPE cartridge; (2) Sample pH is adjusted to > 9 with NaOH and concentrated by freeze-drying; (3) Organic acids are separated by HPLC; (4) Fractions are collected in sealed 12 mL Exetainer® screw capped vials that had been previously spiked with a chemical oxidant and purged with helium; (5) oxidation of the organic acids to CO_2_ is achieved by heating the samples to 90 °C for 15 minutes; (6) the δ^13^C value of the CO_2_ is determined by injection into a gas chromatograph coupled to an isotope ratio mass spectrometer (Agilent 6890 gas chromatograph equipped with a CP Poraplot Q column (27.5 m x 0.32 mm, 10 µm, Varian) maintained at 100 °C, interfaced with a ConFlo IV to a Delta V Plus Mass Spectrometer, both ThermoFisher Scientific); and (7) the Δ^14^C value of the remaining CO_2_ is determined by Accelerator Mass Spectrometry (AMS) at the Institute of Particle Physics of the ETH Zürich using the gas ion source of the 200 kV using a Mini Carbon Dating System (MICADAS)^54^.

### Compound Specific Isotope Analysis of Lipid Biomarkers

Sample H02_072605_Bio1slurpA2_0354 was sequentially extracted three times each with methanol, a 1:1 methanol:dichloromethane mixture, and dichloromethane (DCM). The extracts were combined and the solvent removed under vacuum. The total lipid extract was saponified with 0.5 M KOH in methanol for 2 hours at 70 °C. After cooling, the mixture was back extracted with hexane three times to isolate the neutral fraction, then acidified to pH 2 with 12 M HCl and back extracted three times with hexane:DCM (8:2) to isolate the acid fraction. Identification, quantification, and final checks on compound purity of isolated fractions were performed on a Hewlett Packard 6890 Series gas chromatography system equipped with a mass selective detector (Hewlett Packard 5973).

The acid fraction was methylated overnight at 45 °C with 1.2% HCl in methanol. Phthalic acid of known isotopic composition was simultaneously methylated so that the addition of the –CH_3_ groups could be accounted for in reporting final isotope values. The fatty acid methyl esters (FAMES) were separated over silver impregnated silica gel (AgSiO_2_) with 100% hexane, 100% DCM, and Hexane:ethyl acetate (98:2). Unsaturated fatty acids eluted in the hexane:ethyl acetate fraction and contained primarily (>95%) 16:1 and 18:1, with smaller contributions of other unsaturated fatty acids. This fraction was prepped for radiocarbon analysis without further purification. The DCM fraction contained saturated fatty acids, which were further separated with urea adduction into branched (non-adducted) and unbranched (adducted) compounds^55^. Both fractions were further purified over silica gel to eliminate any traces of urea. The non-adducted fraction contained solely phytanic acid while the adducted fraction contained straight-chain fatty acids, dominated by C16:0. Both fractions were prepped for radiocarbon.

The neutral fraction was passed over columns of activated copper and sodium sulfate to remove sulfur and water, respectively. The neutral fraction was then separated over 5% deactivated silica gel (pore size 60 Å, 40–63 μm particle size, activated at 120 °C for 4 hr). Polar neutral compounds were eluted with DCM:MeOH (1:1) and n-alkanes were eluted with hexane:DCM (9:1). The n-alkane fraction contained saturated and unsaturated pentamethylicosanes (PMIs), squalane, squalene, and variably unsaturated squalenoids. A preparative gas chromatograph was used to further separate these compounds. It consisted of an HP 6890 gas chromatograph with a cooled injection system (Gerstel GmbH, Mülheim an der Ruhr, Germany), a megabore fused silica column (DB-XLB, 30 m × 0.53 mm i.d.), a flame ionization detector, and a preparative fraction collector (PFC, Gerstel). Compounds were collected in glass u-traps attached to the PFC. Collection windows were timed to isolate all of the PMIs, the squalenoids, and squalene. The squalenoid fraction was lost; the squalene fraction was pure and was prepped for radiocarbon. The PMIs contained not only the compounds but also a significant UCM hump. To further purify this fraction the PMIs were first hydrogenated to the fully saturated PMI. The fraction was then passed over Ag-SiO_2_ gel and the hexane fraction was collected. After these steps, the PMI peak contributed 80% of the total peak area.

Two procedural standards, a ^14^C-free C26 alkane (Fluka P/N 52185, lot 405359/1, F^14^C = 0.0016 ± 0.0006) and a modern C30 alkane (Fluka P/N 90270-1 G, lot 044661/1, F^14^C = 0.9918 ± 0.0038) were subjected to similar isolation steps to characterize the contribution of extraneous carbon. For the compounds isolated by AgSiO_2_ chromatography and urea adduction (saturated fatty acids, phytanic acid, unsaturated fatty acids), different concentrations of both process standards were subjected to AgSiO_2_ chromatography, urea adduction, and a final purification over 5% deactivated silica gel. Because the alkane standards will not elute in the same fraction as more polar the compounds of interest, all eluted fractions were combined as one to obtain a maximum representative blank. A separate series of both standards was also injected into the preparative gas chromatograph, trapped, and processed in the same way as the samples. In both cases, the process standards incorporates any extraneous carbon contributed from the vacuum oxidation and introduction into the AMS.

Isolates to be prepped for radiocarbon were dissolved in DCM:MeOH (9:1) and passed over a very short silica gel column directly into pre-combusted (950 °C, 5 hours) quartz tubes. After addition of pre-combusted (950 °C, 5 hours) CuO, the tubes were flame sealed under vacuum and the compounds oxidized to CO_2_ overnight at 950 °C. The CO_2_ was quantified on a vacuum line and transferred to 4 × 70 mm Pyrex tubes for subsequent AMS measurement.

The radiocarbon content was analyzed with a miniaturized radiocarbon dating system (MICADAS)^56^ at the laboratory of Ion Physics of the ETH. Results are reported as F^14^C^57^. Different amounts of radiocarbon-dead phthalic acid and modern oxalic acid (OX-2) were also placed in quartz tubes and prepared in a similar way as the samples and process standards.

The final F^14^C values for the saturated fatty acids, the phytanic acid, and the unsaturated fatty acids were corrected using the extraneous carbon determined by column chromatography and for the addition of the –CH_3_ group. The F^14^C of squalene was corrected for the extraneous carbon determined by prep-GC. In all cases, the extraneous carbon contributed ≤3% of the total C in the sample. Propagated errors from the blank correction and the AMS analysis are reported.

### Metagenomic sequencing

Purified DNA extracted from the two chimney samples was sent to the Josephine Bay Paul Center, Marine Biological Laboratory (MBL) for Illumina shotgun sequencing. Metagenomic libraries were constructed with the Nugen Ultralow Ovation kit according to the manufacturer’s instructions. Paired-end sequencing was conducted with a 100 cycle Illumina HiSeq run. All unassembled sequence data related to this study are available via the SRA identifier SRP049438 and BioProject PRJNA265986. Raw sequence data was processed by the Brazelton lab to trim adapter sequences, remove artificial replicates, and trim reads based on quality so that all reads have a minimum quality score of 25 over a sliding window of 6 bases. Paired-end reads from each chimney sample were assembled separately with Ray Meta^58^ with a kmer of 41. Paired-end reads were subsequently mapped to contigs with fdhA or fdhB sequences, and these reads were re-assembled with a kmer of 61. Several assembly strategies and parameters were tested and compared with MetaQuast^59^ and ALE^60^. The Prokka pipeline^61^ was used for gene prediction and functional annotation. The arguments–metagenome and–proteins were used with Prokka v.1.12 to indicate that genes should be predicted with the implementation of Prodigal v.2.6.2^62^, optimized for metagenomes, and then searched preferentially against a custom protein database as well as the default databases included with Prokka, including Pfam. The database provided was the last free version (2011) of the Kyoto Encyclopedia of Genes and Genomes^63^, obtained from MG-RAST^64^. Contig coverages were calculated by mapping merged reads onto the assembled contigs with Bowtie2 v.2.2.6^65^ and then multiplying the number of mapped reads to each contig by the average read length of 108 base pairs and dividing by the length of the contig. Detailed documentation of all metagenomic data processing is provided on the Brazelton lab’s website (https://baas-becking.biology.utah.edu/data/category/18-protocols), and all custom software and scripts are available at https://github.com/Brazelton-Lab.

### Analyses of formate dehydrogenase genes

Assembled metagenomic contigs with predicted *fdhA* sequences, i.e. those that encode the alpha subunit of formate dehydrogenase (K00123), were identified in each chimney metagenome. The best matches to the predicted *fdhA* genes were identified by conducting a blastx search with each predicted *fdhA* nucleotide sequence against the NCBI non-redundant protein sequences (nr) database. The best match reported in Table 2 of the main text was that which had the highest ‘max score’ in the blastx search. ‘Max score’ was chosen to more heavily weight matches that included the full length of the protein sequence, rather than matches that only had high similarity over a short region of the protein. Reference protein sequences for the phylogeny of archaeal *fdhA* were obtained from all finished archaeal genomes in JGI’s IMG database in July 2016. Predicted *fdhA* protein sequences from the Lost City contigs were included in the tree, and the final tree in Supplemental Figure 2 was refined to include only species with close phylogenetic relationships with Lost City sequences. The multiple sequence alignment was conducted with MUSCLE^66^, and the phylogeny was built using RaxML and the “-f a” option with 100 bootstrap replicates.

### Data Availability

Accession codes can be found in the materials and methods and all computer codes are published and referenced in the text.

## Electronic supplementary material

Supplementary Information
