# Supplementary material for: Deeply-sourced formate fuels sulfate reducers but not methanogens at Lost City hydrothermal field
Source: Sci Rep. 2018 Jan 15;8:755. doi: 10.1038/s41598-017-19002-5 (PMC5768773; doi:10.1038/s41598-017-19002-5)
Supplement: Supplementary file 1 — Supplementary Information [file 41598_2017_19002_MOESM1_ESM.pdf]

Supplementary Information for

Deeply-sourced formate fuels sulfate reducers but not methanogens at Lost City hydrothermal field

Susan Q. Lang[a,b,*], Gretchen L. Früh-Green[b], Stefano M. Bernasconi[b], William J. Brazelton[c], Matthew O. Schrenk[d], Julia M. McGonigle[c]

[a]School of Earth, Ocean and the Environment, University of South Carolina, Columbia, SC, 29208, USA
[b]Department of Earth Sciences, ETH Zürich, Zurich, 8092, Switzerland
[c]Department of Biology, University of Utah, Salt Lake City, UT, 84112, USA
[d]Department of Earth and Environmental Sciences, Michigan State University, East Lansing, MI, 48824, USA

Thermodynamic Calculations

The thermodynamic equilibrium between ΣCO_2 ($CO_{2\ aq}$, H_2CO_3, HCO_3^-, and CO_3^{-2}) and Σformate (HCOOH and $HCOO^-$) was determined at the in situ conditions at each vent, excluding carbonate precipitation and methane generation, taking into account the pH, temperature, and hydrogen content after the conventions of refs. (1) and (2). The measured exit temperature of the fluids from each vent was used instead of an estimated subsurface temperature in order to investigate what processes control the formate content once the fluids are diverted to distinct locations. The *in situ* pH from each location was calculated using Geochemist's Workbench, by taking the fluid chemistry and pH determined at 25°C [3-5] and heating the theoretical fluid to the measured exit temperature[6]. The fluid chemistry from Markers B and 3 are similar enough that they can be plotted on the same line.

Supplementary Figure 1 shows the expected metastable equilibrium speciation between Σformate and ΣCO_2 (excluding carbonate precipitation and methane generation) from Markers 2, C, B, and 3. The actual measured Σformate/ ΣCO_2 values are plotted on the same graph for comparison. From this presentation, it is clear that the amount of Σformate is vastly higher than equilibrium values Markers 2, C, and B. The calculated temperature at which Σformate/ ΣCO_2 came into equilibrium in these locations is <25°C[5], significantly lower than all other estimates of subsurface temperatures as well as the exit fluid temperatures[4,6,7]. The discrepancy between the observed and calculated Σformate/ ΣCO_2 ratios are not due, therefore, to having come to equilibrium in the subsurface and there being insufficient time to re-equilibrate once the fluids exit the seafloor.

In contrast to the other locations, Σformate and ΣCO_2 are close to equilibrium at Marker 3. The calculated temperature at which these two species came into equilibrium is ~75°C is also close to the observed exit temperature of 88°C[5]. The distinction at Marker 3 is that the concentrations of ΣCO_2 are more than an order of magnitude higher than those at Markers 2, C, and B (10-26 versus 0.1 – 0.6 µmol/L)[8]. Markers B and 3 both reside on a single >60 m carbonate monolith called Poseidon, with B lower on the structure. The higher ΣCO_2 concentrations must therefore reflect near-surface entrainment of seawater into Marker 3 fluids, where residual seawater dissolved inorganic carbon (DIC) that has not yet been precipitated as calcium carbonate.

Mixing Calculations and $\delta^{13}C$ values of seawater-derived and mantle-derived formate

The stable carbon and radiocarbon isotope signatures of formate both increase with increasing concentrations between the vent fluids. In the simplest scenario only two sources contribute to the total formate pool: a mantle-derived, $F^{14}C$ free source with a more negative $\delta^{13}C$ signature and an additional modern $F^{14}C$ source with a more positive $\delta^{13}C$ signature. Below, the isotope data is used to constrain the relative concentrations of these two sources, calculate the endmember $\delta^{13}C$ values of the two sources, and determine if this simplistic scenario is robust across the vent field.

The $F^{14}C$ of a total formate pool that is a mixture of these two sources will be:

$$F^{14}C_{total} \times [HCOO^-]_{total} = F^{14}C_{md} \times [HCOO^-]_{md} + F^{14}C_{swd} \times [HCOO^-]_{swd} \quad \text{Eq. 2}$$

where $F^{14}C$ is the radiocarbon isotope value and $[HCOO^-]$ is the concentration of the measured endmember ('total'), the mantle-derived source ('md') and the seawater-derived source ('swd'). The $F^{14}C$ signature of the mantle-derived carbon source is 0. The signature of the seawater-derived carbon is set as the average value of $F^{14}C_{DIC}$ from 800 m and 550 m water depth (1.0225 ± 0.0417)[8].

Applying the measured $F^{14}C_{formate}$ from each location, the contribution of mantle-derived vs. seawater-derived formate is 113 ± 16 vs. 11 ± 11 µmol/L (Marker 2), 109 ± 64 vs. 37 ± 64 µmol/L (Marker B), and 72 ± 8 vs. 86 ± 9 µmol/L (Marker 3). The larger errors in the $F^{14}C_{formate}$ from B results in the partitioning of the sources from this location being poorly constrained.

Again assuming that only two sources contribute to the total formate pool, plotting $\delta^{13}C_{formate}$ vs $1/[HCOO^-]$ for all four sites can used to identify the $\delta^{13}C$ signature of the minor component[9]. The intercept of this line is $\delta^{13}C_{swd} = +8.4 \pm 1.6$ ‰ and represents the signature of the seawater-derived component. The relative contributions of mantle and seawater-derived formate can then be used to determine the $\delta^{13}C$ signature of the major (mantle-derived) component using the mixing relationship of:

$$R_{total} \times [HCOO^-]_{total} = R_{md} \times [HCOO^-]_{md} + R_{swd} \times [HCOO^-]_{swd} \quad \text{Eq. 3}$$

where R is the absolute ratio of $^{13}C/^{12}C$ in a sample or standard, and equivalent to $[\delta^{13}C/1000 + 1] \times R_{stnd}$, and R_{stnd} refers to Vienna Pee Dee Belemnite isotope standard. This approach is best constrained at Marker 2 since the formate pool is dominated by the mantle component and the relative contributions of seawater-derived and mantle-derived formate are available from the $F^{14}C$ data. In this location the calculation results in a $\delta^{13}C_{md}$ of -12.7 ± 3.8 ‰.

In the locations where formate/$\sum CO_2$ are far out of equilibrium (Markers C, 2, B), the concentration of mantle-derived formate is approximately 112 µmol/L, while the concentration of seawater-derived formate increases from 3 to 37 µmol/L. The concentration of mantle-derived formate is substantially lower at Marker 3 (~67 µmol/L), where the formate/$\sum CO_2$ is close to equilibrium.

The stable isotope signature of formate produced from modern carbon (+8.4 ± 1.6‰) is more positive than expected from biological production. Biological isotopic equilibration results in formate that is ~23‰ more negative than the associate dissolved inorganic carbon [10]. The $\sum CO_2$ in Lost City fluids is approximately -9‰ and local seawater DIC is 0.8 ‰ [8], which would result in formate that is -24 to -32‰. Formate produced during the anaerobic degradation of complex organic matter would be more negative than the starting material by 7 - 11‰ [10]. The carbonate-brucite structures through which fluid flows at these locations (Markers 2, B, C, 3) contain high concentrations of complex

organic matter with $\delta^{13}C$ values of -18 to -7‰ [3,7]. Formate produced during the degradation of this material would have an isotopic composition of -29 to -14‰.

The enrichment of ^{13}C in formate derived from modern carbon may be due to its production from, or equilibration with, a dissolved inorganic carbon pool present in only very low concentrations due to its rapid removal by biological update and carbonate precipitation. Calcium carbonate chimneys can reach $\delta^{13}C$ values as high as +13‰, demonstrating that enriched inorganic carbon is present in the field[7]. Biologically derived carbon isolated from Lost City chimneys is consistently more enriched in ^{13}C than typical oceanic organic matter, including bulk biomass from active chimneys ($\delta^{13}C$ = - 12.4 to -3 ‰)[3,7,11], amino acids (-18 to +9 ‰)[12], bacterial lipid biomarkers (-31 to -1.1‰)[11], and archaeal lipid biomarkers (-12 to +24.6 ‰)[11,13]. Given the range of $\delta^{13}C$ biological carbon $\delta^{13}C$ signatures, it is perhaps surprising that a single, modern $\delta^{13}C_{formate}$ could even be identified. The initial $\delta^{13}C$ signature of seawater DIC and the carbonate chimneys is relatively narrow across the field. The stability of the modern $\delta^{13}C_{formate}$ signature may therefore reflect the relative rate at which $\sum CO_2 \rightarrow$ formate proceeds in relation to other removal processes.

The stable isotopes signature of formate derived from mantle carbon (-12.7 ± 3.8 ‰) does not correspond to theoretical calculated isotope equilibrium conditions. At temperatures ranges from 125-225°C, representative of calculated subseafloor temperatures at Lost City, $\delta^{13}C_{formate}$ will be 14-18 ‰ more negative than the $\delta^{13}C_{CO2}$ [14]. Fluid inclusions in primitive olivine have $\delta^{13}C_{CO2}$ values of -12 to -2 ‰ [15,16] which would result in $\delta^{13}C_{formate}$ ~ -30 to -16 ‰. The stable isotopes of methane (-13.6 to -9.3 ‰) and short-chain hydrocarbons (-16.9 to -13.1‰) are also far more positive than their theoretical equilibrium values, which has been attributed to near-quantitative reduction of mantle CO_2 to hydrocarbons[8]. A similar process could control the isotope signature of mantle-derived formate.

Acetate $\delta^{13}C$ values

The isotopic composition of acetate was also obtained for the same samples (Main Text Table 1; Supplemental Table 1). The average $\delta^{13}C_{acetate}$ from the four individual locations ranged from -26.6‰ to -20.3‰ and overall averaged -23.6 ± 4.7 ‰. Replicates from sample 3874p22 from Marker C were extremely reproducible (-26.7 ± 1.0‰, n=3) while samples from Marker B had much higher variability. While the average isotopic composition of samples from B was -23.2 ± 6.0‰, one sample (3875m23) had the most enriched composition, with a $\delta^{13}C_{acetate}$ = -16.5‰. In contrast, a replicate from a separate sample from Marker B (3875m24) had the most depleted composition, yielding $\delta^{13}C_{acetate}$ = -34.0‰. At this time the underlying causes of this variability are unknown.

The isotopic composition of acetate could be attributed to either a combination of anaerobic fermentation and acetogenesis or, possibly, the thermocatalytic breakdown of complex organics. Acetate is a primary by-product of anaerobic microbial fermentation of complex organic matter. At Lost City, $\delta^{13}C$ of the organic matter in the carbonate chimneys from the actively venting chimneys that were sampled for $\delta^{13}C_{acetate}$ ranges

from -18 to -7‰ [3,7]. Assuming a fractionation factor of 0 to +3 ‰ [10], acetate produced from anaerobic fermentation from this material would have an isotopic signature of -18 to -4‰. Acetate produced from autotrophic acetogenesis ($2HCO_3^- + 4H_2 + H^+ \rightarrow CH_3COO^- + 4\ H_2O$) has a $\delta^{13}C$ -60 to -54‰ lighter than the starting HCO_3^- [17,18]. The best estimate of the isotopic signature of $\sum CO_2$ at Lost City is ≈-9‰ [8] which would result in acetate with an isotopic signature of -69 to -63‰. One possibility is that therefore that acetate is produced from a combination of these two sources, resulting in a mixed isotopic signature of ~ -24‰.

Alternatively, complex organic matter subjected to elevated temperatures can break down thermocatalytically. Methane is the most widely recognized product of this process, and requires high temperatures to promote the fission of carbon-carbon bonds[19] but the heating of sediments and kerogen also results in high concentrations of organic acids, dominated by acetate[20-27]. The apparent activation energy required to produce acetate is less than a third of that required for methane and other hydrocarbons[22,28,29], thus acetate production can be initiated at temperatures lower than methane production. At the lowest temperature typically used for aqueous pyrolysis experiments (200ºC) acetate is readily produced[22,23,30].

It is unclear if the temperatures of the studied chimneys (vent temperatures 55-91ºC) would be high enough to promote the thermocatalytic production of acetate within the towers. Few experiments investigating these abiotic processes have been carried out at lower temperatures due to slower reaction times and concerns with biological interference. Parkes et al. (2011)[31] reported detectable acetate when sterilized sediments were heated to temperatures as low at 40ºC, although the authors suggest this release may have partially been an artifact of prior autoclaving at 121ºC. Elevated acetate concentrations have also been attributed to thermal alteration in pore fluids from Guaymas Basin, where maximum temperatures ranged from 80°C-125°C[26], and in formation waters in reservoirs with temperatures 90°C-250°C [25]. It is important to note that in laboratory pyrolysis experiments[20,22-24] and in field measurements[25,26], formic acid was rarely detected and, when it was, its concentration was typically one tenth that of acetate. Therefore while elevated acetate concentrations could potentially be attributed to thermal alteration, the same is not true for formate.

Acetate produced during hydrous pyrolysis at temperatures <250°C had an isotopic signature that was depleted in ^{13}C by 0 – 3 ‰ compared to the starting kerogen[26,30]. This fractionation factor is complicated, however, by the partial equilibration of the carboxyl carbon with DIC after the acetate was generated. If the alkyl carbon can be used as representative of the initial acetate, its $\delta^{13}C$ is depleted by ~5-6‰ compared to the starting kerogen[30]. Given the low ΣCO_2 concentrations in Lost City fluids[8], an equilibration between the carboxyl carbon of acetate and DIC is probably limited in endmember fluids, although it may occur to some extent after mixing with seawater. Complex organic matter in these chimneys has $\delta^{13}C$ values of -18 to -7‰ [3,7] which would result in $\delta^{13}C_{acetate} \approx$ -24 to -12‰. Because equilibrium isotope effects are magnified at lower temperatures[32], the $\delta^{13}C_{acetate}$ may be drawn to more depleted values. Therefore, the

observed isotopic composition of acetate would also be consistent with the thermal breakdown of chimney biomass.

References

1 McCollom, T. M. & Seewald, J. S. Experimental constraints on the hydrothermal reactivity of organic acids and acid anions: I. Formic acid and formate. *Geochimica Et Cosmochimica Acta* **67**, 3625-3644, doi:10.1016/s0016-7037(03)00136-4 (2003).

2 Seewald, J. S., Zolotov, M. Y. & McCollom, T. Experimental investigation of single carbon compounds under hydrothermal conditions. *Geochimica Et Cosmochimica Acta* **70**, 446-460, doi:10.1016/j.gca.2005.09.002 (2006).

3 Lang, S. Q. *et al.* Microbial utilization of abiogenic carbon and hydrogen in a serpentinite-hosted system. *Geochimica Et Cosmochimica Acta* **92**, 82-99, doi:10.1016/j.gca.2012.06.006 (2012).

4 Proskurowski, G., Lilley, M. D., Kelley, D. S. & Olson, E. J. Low temperature volatile production at the Lost City Hydrothermal Field, evidence from a hydrogen stable isotope geothermometer. *Chemical Geology* **229**, 331-343, doi:10.1016/j.chemgeo.2005.11.005 (2006).

5 Lang, S. Q., Butterfield, D. A., Schulte, M., Kelley, D. S. & Lilley, M. D. Elevated concentrations of formate, acetate and dissolved organic carbon found at the Lost City hydrothermal field. *Geochimica Et Cosmochimica Acta* **74**, 941-952, doi:10.1016/j.gca.2009.10.045 (2010).

6 Seyfried, W. E., Pester, N. J., Tutolo, B. M. & Ding, K. The Lost City hydrothermal system: Constraints imposed by vent fluid chemistry and reaction path models on subseafloor heat and mass transfer processes. *Geochimica Et Cosmochimica Acta* **163**, 59-79, doi:10.1016/j.gca.2015.04.040 (2015).

7 Kelley, D. S. *et al.* A serpentinite-hosted ecosystem: The lost city hydrothermal field. *Science* **307**, 1428-1434, doi:10.1126/science.1102556 (2005).

8 Proskurowski, G. *et al.* Abiogenic hydrocarbon production at Lost City hydrothermal field. *Science* **319**, 604-607, doi:10.1126/science.1151194 (2008).

9 Keeling, C. D. The concentration and isotopic abundances of carbon dioxide in rural and marine air. *Geochimica Et Cosmochimica Acta* **24**, 277-298, doi:10.1016/0016-7037(61)90023-0 (1961).

10 Penning, H. & Conrad, R. Carbon isotope effects associated with mixed-acid fermentation of saccharides by Clostridium papyrosolvens. *Geochimica Et Cosmochimica Acta* **70**, 2283-2297, doi:10.1016/j.gca.2006.01.017 (2006).

11 Bradley, A., Hayes, J. & Summons, R. Extraordinary C-13 enrichment of diether lipids at the Lost City Hydrothermal Field indicates a carbon-limited ecosystem. *Geochimica Et Cosmochimica Acta* **73**, 102-118, doi:10.1016/j.gca.2008.10.005 (2009).

12 Lang, S. Q., Fruh-Green, G. L., Bernasconi, S. M. & Butterfield, D. A. Sources of organic nitrogen at the serpentinite-hosted Lost City hydrothermal field. *Geobiology* **11**, 154-169, doi:10.1111/gbi.12026 (2013).

13 Mehay, S. *et al.* Record of archaeal activity at the serpentinite-hosted Lost City Hydrothermal Field. *Geobiology* **11**, 570-592, doi:10.1111/gbi.12062 (2013).

14 Galimov, E. M. Carbon isotopes in oil-gas geology. *Carbon isotopes in oil-gas geology NASA Transl. into English of the book "Izotopy Ugleroda v Neftergazovy Geologii" Moscow, Nedra Press, 1973, p 1-384* (1975).

15 Kelley, D. S. & Fruh-Green, G. L. Abiogenic methane in deep-seated mid-ocean ridge environments: Insights from stable isotope analyses. *Journal of Geophysical Research-Solid Earth* **104**, 10439-10460, doi:10.1029/1999jb900058 (1999).
16 Kelley, D. S. & Fruh-Green, G. L. Volatile lines of descent in submarine plutonic environments: Insights from stable isotope and fluid inclusion analyses. *Geochimica Et Cosmochimica Acta* **65**, 3325-3346, doi:10.1016/s0016-7037(01)00667-6 (2001).
17 Gelwicks, J. T., Risatti, J. B. & Hayes, J. M. Carbon isotope effects associated with autotrophic acetogenesis. *Organic Geochemistry* **14**, 441-446, doi:10.1016/0146-6380(89)90009-0 (1989).
18 Preuss, A., Schauder, R., Fuchs, G. & Stichler, W. Carbon isotope fractionation by autotrophic bacteria with 3 different CO2 fixation pathways. *Zeitschrift Fur Naturforschung C-a Journal of Biosciences* **44**, 397-402 (1989).
19 Tissot, B. P. & Welte, D. H. *Petroleum Formation and Occurrence.* (Springer, 1984).
20 Cooles, G. P., Mackenzie, A. S. & Parkes, R. J. Non-hydrocarbons of significance in petroleum-exploration - volatile fatty-acids and non-hydrocarbon gases. *Mineralogical Magazine* **51**, 483-493, doi:10.1180/minmag.1987.051.362.03 (1987).
21 Carothers, W. W. & Kharaka, Y. K. Aliphatic acid anions in oil-field waters - implications for origin of natual gas. *Aapg Bulletin-American Association of Petroleum Geologists* **62**, 2441-2453 (1978).
22 Barth, T., Borgund, A. E. & Hopland, A. L. Generation of organic-compounds by hydrous pyrolysis of kimmeridge oil-shale - bulk results and activation energy calculations. *Organic Geochemistry* **14**, 69-76, doi:10.1016/0146-6380(89)90020-x (1989).
23 Kawamura, K., Tannenbaum, E., Huizinga, B. J. & Kaplan, I. R. Volatile organic acids generated from kerogen during laboratory heating. *Geochemical Journal* **20**, 51-59 (1986).
24 Eglinton, T. I., Curtis, C. D. & Rowland, S. J. Generation of water-soluble organic acids from kerogen during hydrous pyrolysis - implications for porosity development. *Mineralogical Magazine* **51**, 495-503, doi:10.1180/minmag.1987.051.362.04 (1987).
25 Fisher, J. B. Distribution and occurrence of aliphatic acid anions in deep subsurface waters. *Geochimica Et Cosmochimica Acta* **51**, 2459-2468, doi:10.1016/0016-7037(87)90297-3 (1987).
26 Martens, C. S. Generation of short chain organic-acid anions in hydrothermally altered sediments of the Guaymas Basin, Gulf of California. *Applied Geochemistry* **5**, 71-76 (1990).
27 Seewald, J. S., Seyfried, W. E. & Thornton, E. C. Organic-rich sediment alteration - an experimental and theoretical study at elevated temperatures and pressures. *Applied Geochemistry* **5**, 193-209 (1990).
28 Barth, T. & Nielsen, S. B. Estimating kinetic-parameters for generation of petroleum and single compounds from hydrous pyrolysis of source rocks. *Energy & Fuels* **7**, 100-110, doi:10.1021/ef00037a017 (1993).

29 Knauss, K. G., Copenhaver, S. A., Braun, R. L. & Burnham, A. K. Hydrous pyrolysis of New Albany and Phosphoria Shales: production kinetics of carboxylic acids and light hydrocarbons and interactions between the inorganic and organic chemical systems. *Organic Geochemistry* **27**, 477-496, doi:10.1016/s0146-6380(97)00081-8 (1997).

30 Dias, R. F., Freeman, K. H., Lewan, M. D. & Franks, S. G. delta C-13 of low-molecular-weight organic acids generated by the hydrous pyrolysis of oil-prone source rocks. *Geochimica Et Cosmochimica Acta* **66**, 2755-2769, doi:10.1016/s0016-7037(02)00871-2 (2002).

31 Parkes, R. J. *et al.* Prokaryotes stimulate mineral H-2 formation for the deep biosphere and subsequent thermogenic activity. *Geology* **39**, 219-222, doi:10.1130/g31598.1 (2011).

32 Hayes, J. M. Fractionation of carbon and hydrogen isotopes in biosynthetic processes. *Stable Isotope Geochemistry* **43**, 225-277, doi:10.2138/gsrmg.43.1.225 (2001).

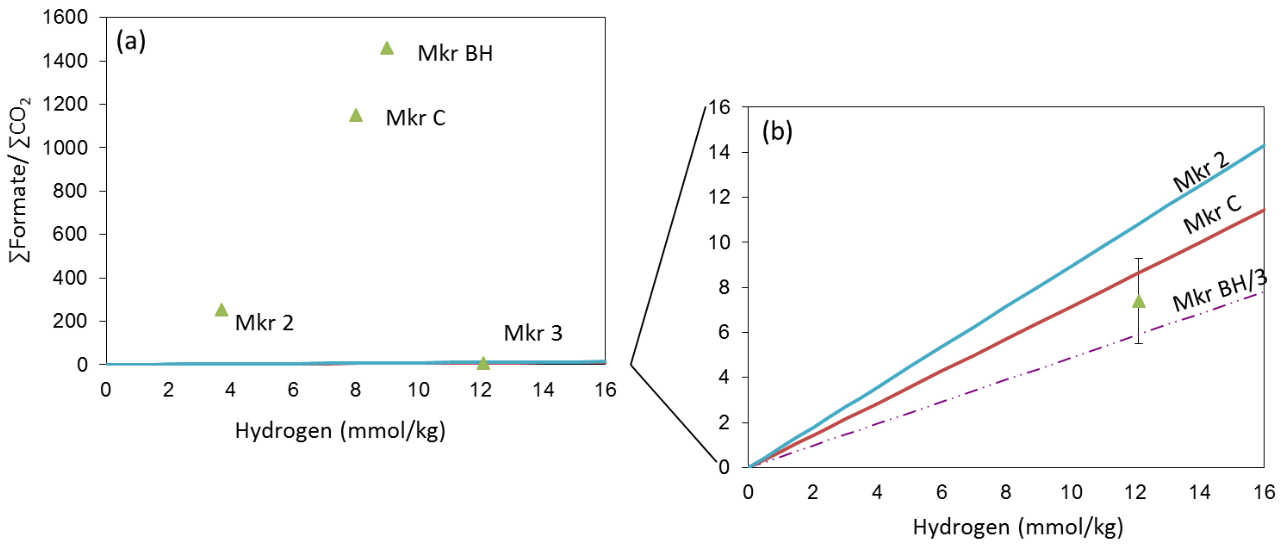

Supplementary Figure 1. Calculated equilibrium between ∑Formate/∑CO_2 vs H_2 at Markers 2, C, B, and 3 (lines) compared to observations (triangles). Equilibration lines in the upper left plot are not distinguishable from the x-axis; note the y-axis scale difference with the lower right plot.

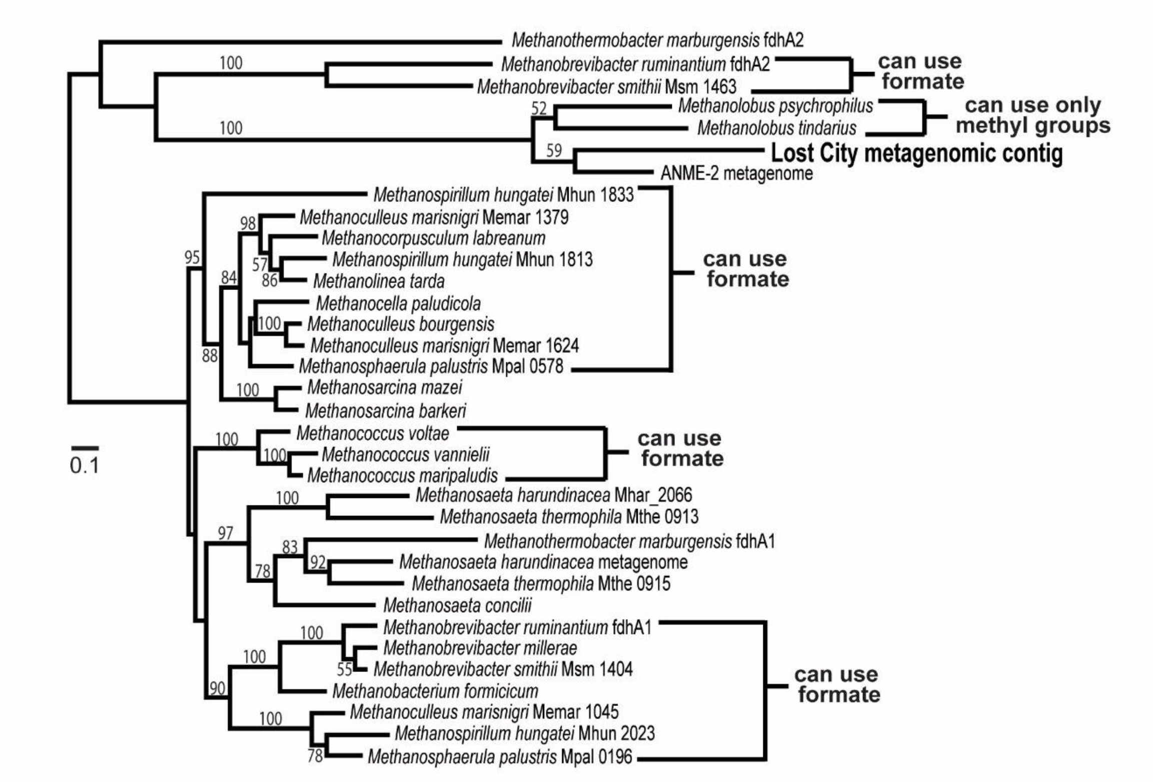

Supplementary Figure 2. Phylogenetic tree of *fdhA* (formate dehydrogenase alpha subunit) predicted protein sequences showing the relationship between a Lost City metagenomic sequence and methanogen *fdhA* sequences. Distinctions between multiple copies in the same genome are provided where necessary. Accession numbers for all sequences on the tree are provided in the Materials and Methods.

Supplemental Table S1. Isotope values of formate and acetate from Lost City fluids

Sample	Location	Mg	E.M. Formate	E.M. Acetate	$\delta^{13}C_{Formate}$		$\delta^{13}C_{Acetate}$		$F^{14}C_{Formate}$	
	(Marker)	(mmol/kg)	(μmol/L)[1]	(μmol/L)[a]	(‰)	(± 1σ)	(‰)	(± 1σ)	($F^{14}C$)[b]	(± 1σ)[c]
3874p22	C	14.1	115 ± 16	8 ± 0.4	-11.2	0.8	-26.6	0.5		
3874p22	C	14.1	115 ± 16	8 ± 0.4	-12.6	1.2	-25.8	0.5		
3874p22	C	14.1	115 ± 16	8 ± 0.4	-13.0	0.7	-27.8	0.5		
3874p22	C	14.1	115 ± 16	8 ± 0.4	-11.5	0.9	bdl			
3874bf17	C	12.3	115 ± 16	8 ± 0.4	-12.1	1.2	-26.2	0.7	bdl	
3870bf16	2	8.2	124 ± 12	10 ± 1	-12.6	0.5	-21.7	0.6	0.094	0.092
3870bf16	2	8.2	124 ± 12	10 ± 1	-8.9	1.2	-19.0	1.0	bdl	
3876m23	B	2.1	146 ± 7	8 ± 0.4	-8.6	0.9	-24.5	0.7	0.247	0.058
3876m23	B	2.1	146 ± 7	8 ± 0.4	-4.3	1.6	-19.9	1.1		
3876m23	B	2.1	146 ± 7	8 ± 0.4	-6.1	1.3	-18.4	1.4	0.138	0.054
3875m24	B	3.0	146 ± 7	8 ± 0.4	-9.1	0.6	-27.4	0.7	0.218	0.039
3875m24	B	3.0	146 ± 7	8 ± 0.4	-7.9	0.7	-34.0	1.1		
3875m23	B	3.0	146 ± 7	8 ± 0.4	-8.3	1.2	-16.5	1.1	bdl	
3874b19	B	1.1	146 ± 7	8 ± 0.4	-6.7	0.8	-21.7	0.8	0.428	0.121
3862m11	3	8.1	158 ± 16	4 ± 0.4	-6.8	2.1	-21.2	0.9	0.556	0.129

[a]Average concentration and standard deviation of multiple samples from ref. 5

[b] Corrected for AMS instrument blank using calibrated gasses and an extraneous blank contribution.

[c] Error includes counting statistics and corrections for AMS instrument as well as the propagated error from an extraneous blank with $F^{14}C = 1.0 \pm 0.2$ and C = 1.1 ± 0.2 μg C

Table S2. Mixing calculation to identify the contribution from seawater (sw) and mantle-derived formate

**Location (Marker)**	**E.M. Formate (μmol/L)[a]**	**$\delta^{13}C_{Formate}$ (‰)**	**(± 1σ)**	**$F^{14}C_{Formate}$ ($F^{14}C$)[b]**	**(± 1σ)**	**SW-derived (μmol/L)[c]**	**Mantle-derived (μmol/L)[c]**
C	115 ± 16	-12.1	0.7	bdl			
2	124 ± 12	-10.8	2.6	0.094	0.092	11 ± 11	113 ± 16
B	146 ± 7	-7.3	1.7	0.257	0.122	37 ± 64	109 ± 64
3	158 ± 16	-6.8	2.1	0.556	0.129	86 ± 9	72 ± 8

[a] Ref. (5)

[b] Calculated average and standard deviation of all analyses for each location

[c] Assumes two endmember mixing, with seawater derived component with a $F^{14}C$ of 1.0225 ± 0.0417 [8] and a mantle derived component having a $F^{14}C$ of 0.
